# Peritoneal hydatidosis secondary to ruptured hepatic hydatid cyst—a rare presentation: a case report

**DOI:** 10.1093/jscr/rjaf005

**Published:** 2025-02-04

**Authors:** Ashenafi Amsalu Feleke, Desiybelew Chanie Mekonnen, Meron Berhanu Zeneber, Muluken Assefa Zemariam, Gebrehiwot Aderaw Workneh, Asratu Getnet Amare

**Affiliations:** Department of Surgery, School of Medicine, College of Medicine and Health Sciences, University of Gondar, Maraki Street, Central Gondar Zone, P.O. Box 196, Gondar 6200, Ethiopia; Department of Surgery, School of Medicine, College of Medicine and Health Sciences, University of Gondar, Maraki Street, Central Gondar Zone, P.O. Box 196, Gondar 6200, Ethiopia; Department of Surgery, School of Medicine, College of Medicine and Health Sciences, University of Gondar, Maraki Street, Central Gondar Zone, P.O. Box 196, Gondar 6200, Ethiopia; Department of Surgery, School of Medicine, College of Medicine and Health Sciences, University of Gondar, Maraki Street, Central Gondar Zone, P.O. Box 196, Gondar 6200, Ethiopia; Department of Surgery, School of Medicine, College of Medicine and Health Sciences, University of Gondar, Maraki Street, Central Gondar Zone, P.O. Box 196, Gondar 6200, Ethiopia; Department of Surgery, School of Medicine, College of Medicine and Health Sciences, University of Gondar, Maraki Street, Central Gondar Zone, P.O. Box 196, Gondar 6200, Ethiopia

**Keywords:** *E. granulosus*, pericystectomy, hydatid cyst, rupture, case report

## Abstract

Peritoneal hydatidosis (PH) secondary to rupture of hepatic cyst is an exceedingly rare clinical entity. We present a case of a 25-year-old male patient with PH with a hepatic hydatid cyst. The patient underwent laparotomy, total pericystectomy, segmentectomy, and cholecystectomy with a favorable postoperative course. This case highlights the challenges in diagnosing and managing these rare conditions. This is the first reported case in Ethiopia.

## Introduction

Hydatid disease is a significant public health issue in Asia, the Mediterranean, South America, and Africa. With increasing immigration, the prevalence of the disease has also risen in Europe and North America [1].

Among the four recognized species of *Echinococcus*, *Echinococcus. granulosus* is the primary cause of hydatidosis in humans [2, 3]. The liver (65%) and lungs (25%) are the most common sites of primary infection caused by *E. granulosus*. Secondary infection may occur following cyst rupture, allowing scolices to grow in the peritoneum [1]. Rupture can occur spontaneously or as a result of trauma, typically from increased pressure within the cystic fluid [4, 5]. Predisposing factors for cyst rupture include multiple hydatid cysts, cyst location in segment VI of the liver [6], young age, cyst diameter >10 cm, and superficial cyst location [7].

This case report describes an illustrative instance of peritoneal hydatidosis (PH) secondary to liver hydatid cyst rupture and provides a detailed account of its unique management.

## Case presentation

A 25-year-old male patient was referred to our tertiary hospital with complaints of worsening abdominal swelling over the past 2 weeks, a condition that began in childhood. He reported a history of intermittent abdominal cramps, shortness of breath, and early satiety. Since the age of 12, he had experienced repeated admissions to a primary hospital and had undergone peritoneal tapping twice per month, attributed to suspected ascites secondary to chronic liver disease. He also reported difficulty lying supine.

**Figure 1:**
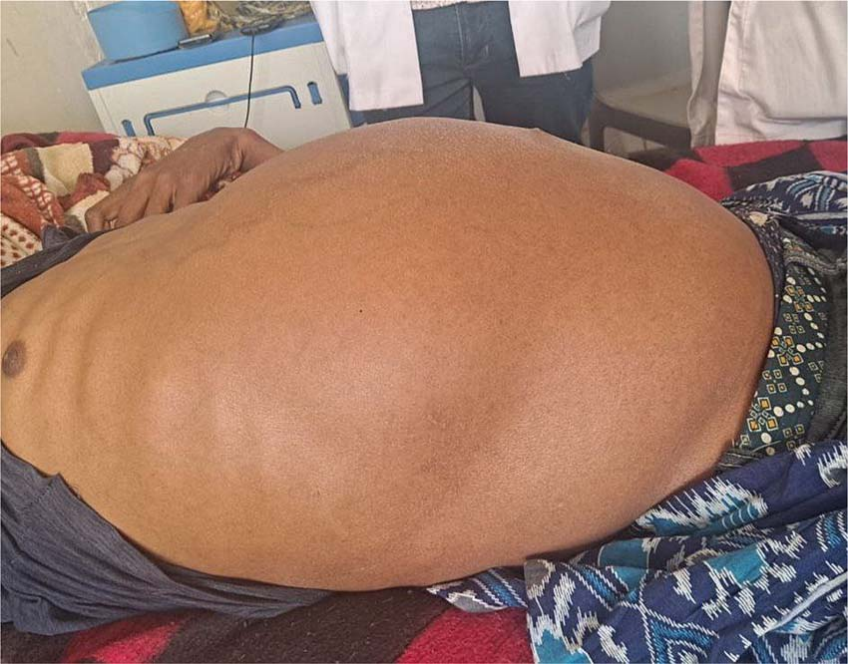
Picture showing hugely distended abdomen.

On examination, the patient appeared chronically ill. His vital signs were in normal range except for tachypnea of 48 breaths per minute. Chest examination revealed decreased air entry over the lower posterior one-third of the right lung field. Abdominal examination revealed a grossly distended abdomen (Fig. 1) with a palpable, firm, and non-tender mass measuring 20 × 30 cm.

Laboratory analysis revealed an alkaline phosphatase level elevated to five times the normal limit. Other blood test results were within normal ranges.

An abdominal ultrasound revealed a large multicystic intra-abdominal mass with peripheral calcification, displacing the liver superiorly as well as the spleen and bowel loops anterolaterally. Additionally, another hypoechoic mass measuring 2 × 1.5 cm with peripheral enhancement was identified in segment VI of the liver.

An abdominal CT scan demonstrated a massive non-enhancing cystic mass with a thick rim of peripheral calcification, measuring 21 × 28 cm, containing peripherally located daughter cysts and pushing the gallbladder anteriorly. Another well-defined, thin-walled, large peritoneal cystic mass, and measuring 13 × 24 × 28 cm, was observed. This mass exhibited fluid attenuation and a focal anteroposterior wall defect, closely apposed to the hepatic cystic mass. It displaced bowel loops, great vessels, and the right kidney posteroinferiorly while mediating the pancreas.

**Figure 2:**
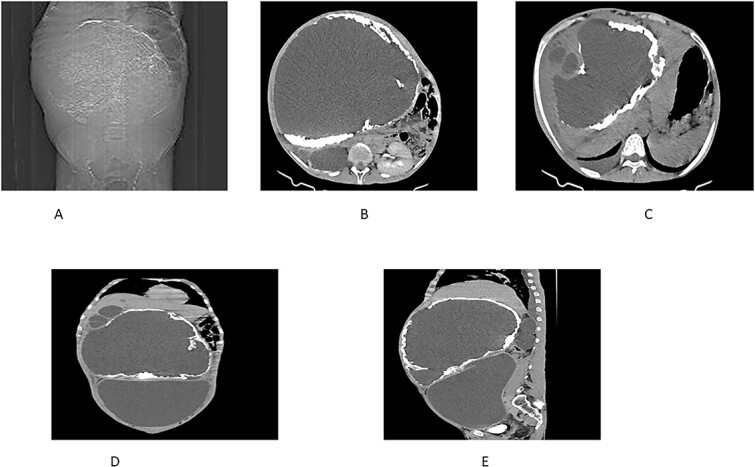
(A) Scout view showing a large intra-abdominal mass with peripheral calcification. (B) Axial view highlighting a hepatic cyst and an atrophied right kidney with a thinned-out cortex. (C) Axial view depicting a right pleural fluid collection, a hepatic cyst, and two active daughter cysts. (D, E) Coronal and sagittal views depicting the hepatic cyst and the peritoneal cyst (white cross).

Further findings included pericalyceal dilatation of the right kidney with an atrophied and thinned-out cortex. Additionally, there was a fluid-attenuating collection measuring ~2.3 cm in depth within the right pleural cavity (Fig. 2).

**Figure 3:**
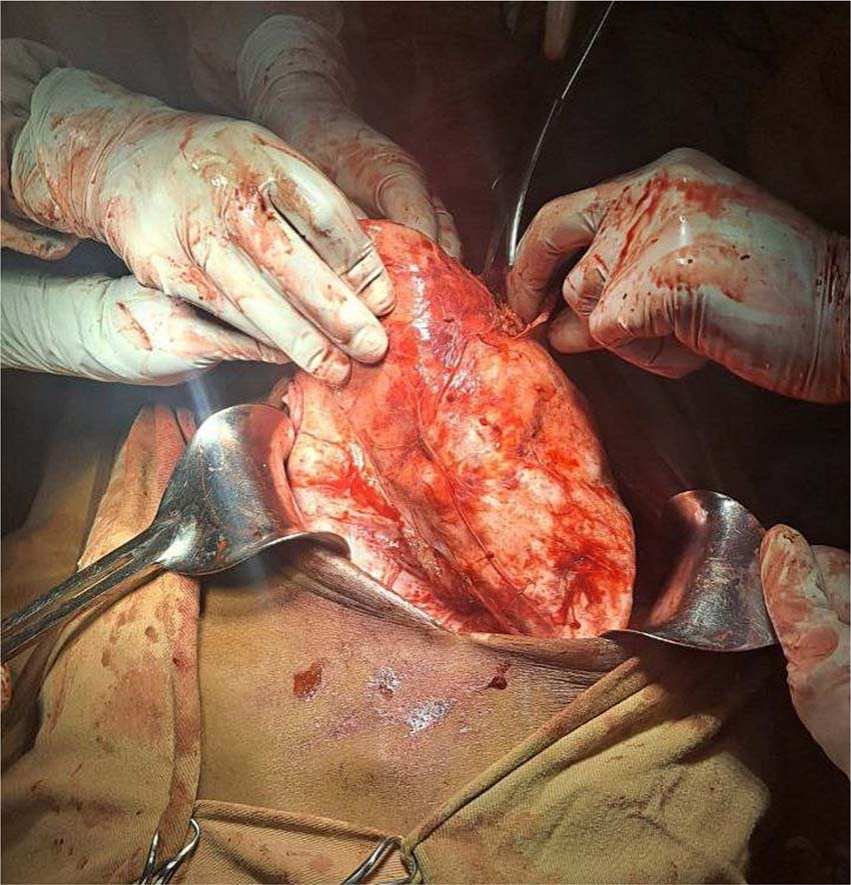
A large intrahepatic mass communicating with a massive peritoneal cystic mass.

**Figure 4:**
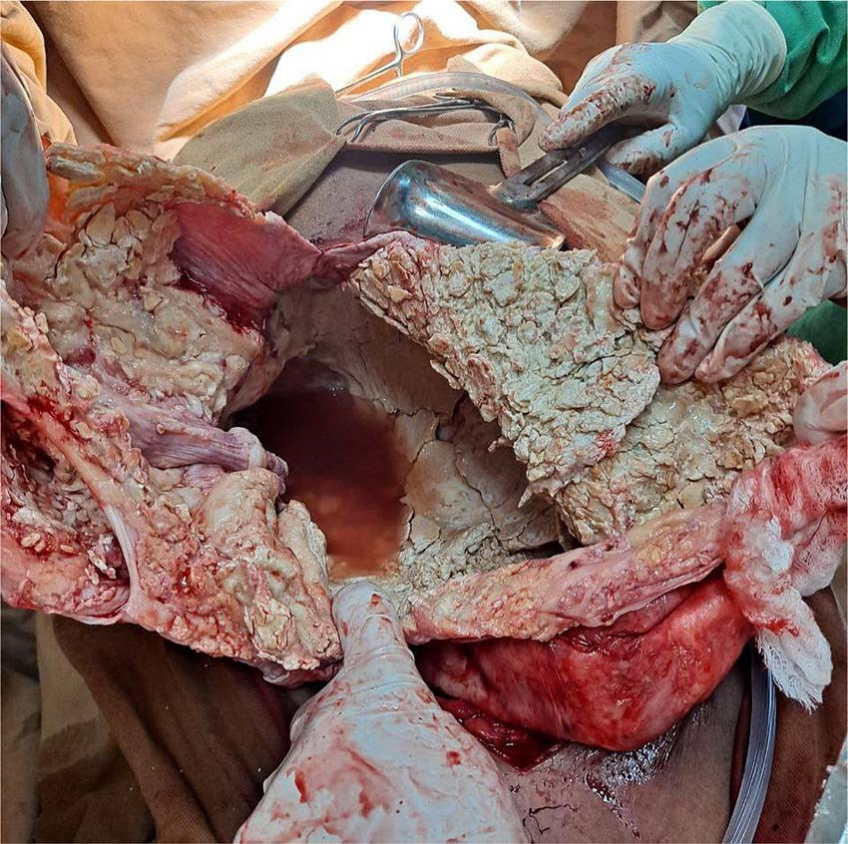
A calcified capsule with evacuated hydatid debris.

The surgical operation was planned as a semi-elective laparotomy. A midline incision was made, revealing a 25 × 25 cm cystic mass with a calcified wall located at segments IVB, V, and VII of the liver, pushing against and attached to the gallbladder. This mass communicated with another peritoneal cystic mass measuring 20 × 20 cm, containing 20 liters of fluid and hydatid sand, and displacing the entire bowel to the left (Figs 3 and 4). Segment VI of the liver was thinned out and contained two additional active cysts. The right kidney was atrophied and displayed a ‘water bag’ appearance. The peritoneal surface and diaphragm were calcified, and adhesions were observed between the intra-abdominal cyst, abdominal wall, and urinary bladder. About 96% alcohol was used as a sclerosing agent.

A total pericystectomy, cholecystectomy, and segmental resection of segment VI with the active cysts were performed. A drain was placed in the peritoneal cavity.

Intraoperatively, the patient developed shock, requiring the initiation, and gradual escalation of a low dose of noradrenaline. Postoperatively, he was transferred to the surgical intensive care unit for further management. Within 24 hours, his shock resolved, noradrenaline was tapered and discontinued, and he underwent a pleural tap, yielding 400 ml of serous fluid with non-revealing pleural fluid analysis. The chest condition subsequently improved, and the patient was extubated. By the fourth postoperative day, he was transferred to the recovery unit.

Postoperative albendazole therapy was initiated for a planned duration of six months. The patient was discharged from the hospital 10 days after surgery. He returned to the surgical referral clinic a week later, where the drain was removed.

## Discussion

*E. granulosus* is hyperendemic in Africa, particularly in Ethiopia [8]. Primary PH is rare, accounting for only 2% of all intra-abdominal hydatidosis cases. It may remain asymptomatic for a period or present with symptoms caused by the pressure effects of the cyst [9].

PH is a parasitic condition caused by the seeding of the peritoneal serosa by *E. granulosus* larvae [10]. It is often secondary to the rupture or fissuring of hepatic hydatid cysts, representing ~5%–16% of all hydatid cyst locations. Spontaneous rupture occurs in 78% of cases and is favored by factors such as superficial cyst location, large size, thin wall, and high intracystic pressure. The stages following rupture reflect the reaction of the peritoneal serosa to the hydatid aggression. These can present as simple hydatid ascites when the cyst is monolocular, free hydatids in the large peritoneal cavity when the cyst is multivesicular, or hydatid peritonitis if the cyst contents are infected [7].

Rupture of hydatid cysts is classified into three types according to Lewall and McCorkell: contained, communicating, and direct. Contained rupture occurs when only the endocyst ruptures and the cyst contents remain within the pericyst derived from the host. Communicating rupture happens when the cyst contents escape through biliary or bronchial radicles incorporated into the pericyst. Direct rupture occurs when both the endocyst and pericyst tear, releasing cyst contents directly into the peritoneal or pleural cavities or other structures [11].

The symptoms of PH depend on the size and number of cysts. It may be asymptomatic or present with non-specific symptoms such as abdominal pain, fullness, vomiting, anorexia, and dyspepsia [12]. In our patient, the symptoms included abdominal pain, early satiety, and fullness.

Diagnosis is typically made via ultrasound or computed tomography (CT) scan. CT is the preferred imaging modality for peritoneal disease. Serological tests are also used in diagnosis, though their sensitivity and specificity vary [13]. In our case, both ultrasound and CT were suggestive, but serologic tests were not performed.

The mainstay of treatment for PH is surgery, either open or laparoscopic. In some cases, complete removal of the peritoneal lesions is not feasible. Therefore, we use adjunct medications like albendazole, mebendazole, and praziquantel to minimize the risk of residual parasitic cysts and recurrence [12].

## Conclusion

PH is a rare condition, typically secondary to liver hydatid cysts. Diagnosis relies on imaging, while surgical intervention remains the primary treatment. A high index of suspicion, accurate diagnostic evaluation, and timely management are essential to prevent complications and achieve favorable outcomes. This case underscores the critical importance of thorough history-taking in every patient encounter.

## References

[1] Khuroo MS . Hydatid disease: current status and recent advances. Ann Saudi Med 2002;22:56–64. 10.5144/0256-4947.2002.56. 17259768

[2] Anadol D, Özçelik U, Kiper N, et al. Treatment of hydatid disease. Paediatr Drugs 2001;3:123–35. 10.2165/00128072-200103020-00005. 11269639

[3] Thompson R . The Biology of Echinococcus and Hydatid Disease. 1986.

[4] Limeme M, Yahyaoui S, Zaghouani H, et al. Spontaneous intraperitoneal rupture of hepatic hydatid cyst: a rare cause of ascites. BMC Surg 2014;14:1–4. 10.1186/1471-2482-14-99. 25427421 PMC4256059

[5] Toumi O, Noomen F, Salem R, et al. Intraperitoneal rupture of hydatid cysts. Eur J Trauma Emerg Surg 2017;43:387–91. 10.1007/s00068-016-0662-9. 27084544

[6] Dirican A, Yilmaz M, Unal B, et al. Ruptured hydatid cysts into the peritoneum: a case series. Eur J Trauma Emerg Surg 2010;36:375–9. 10.1007/s00068-009-9056-6. 26816043

[7] Fatine A, Eddaoudi Y, Bouali M, et al. Peritoneal hydatidosis: an exceptional case report. Ann Med Surg 2022;83. 10.1016/j.amsu.2022.104606. PMC9661632 36389191

[8] Fuller GK, Fuller DC. Hydatid disease in Ethiopia: clinical survey with some immunodiagnostic test results. Am J Trop Med Hyg 1981;30:645–52. 10.4269/ajtmh.1981.30.645. 7258484

[9] Hegde N, Hiremath B. Case report: primary peritoneal hydatidosis. BMJ Case Rep 2013;2013:bcr2013200435. 10.1136/bcr-2013-200435. PMC3762414 23912661

[10] Eckert J, Deplazes P. Biological, epidemiological, and clinical aspects of echinococcosis, a zoonosis of increasing concern. Clin Microbiol Rev 2004;17:107–35. 10.1128/CMR.17.1.107-135.2004. 14726458 PMC321468

[11] Adhikari S, Bhattarai M, Gyawali S, et al. Acute abdomen due to rupture of a hydatid cyst of the liver: a rare complication–a case report. Annals of Medicine and Surgery 2023;85:1172–6. 10.1097/MS9.0000000000000383. 37113932 PMC10129216

[12] Trajkovski G, Antovic S, Kostovski O, et al. Hydatid cysts of the liver with concomitant massive peritoneal hydatidosis: a case report. Radiology Case Reports 2022;17:2394–9. 10.1016/j.radcr.2022.04.008. 35570874 PMC9096448

[13] Acharya AN, Gupta S. Peritoneal hydatidosis: a review of seven cases. Trop Gastroenterol 2010;30:32–4. 19624085

